# Duplication and diversification of lectin receptor-like kinases (*LecRLK*) genes in soybean

**DOI:** 10.1038/s41598-018-24266-6

**Published:** 2018-04-12

**Authors:** Ping-Li Liu, Yuan Huang, Peng-Hao Shi, Meng Yu, Jian-Bo Xie, LuLu Xie

**Affiliations:** 1 College of Biological Sciences and Biotechnology, Beijing Forestry University, Beijing, 100083 China; 2 Institute of Hutchison Whampoa Guangzhou Baiyunshan Chinese Medicine Co., Ltd, Guangzhou, 510515 China; 3 Department of Chinese Cabbage, Chinese Academy of Agricultural Sciences, Beijing, 100081 China

## Abstract

Lectin receptor-like kinases (LecRLKs) play important roles in plant development and stress responses. Although genome-wide studies of LecRLKs have been performed in several species, a comprehensive analysis including evolutionary, structural and functional analysis has not been carried out in soybean (*Glycine max*). In this study, we identified 185 putative *LecRLK* genes in the soybean genome, including 123 G-type, 60 L-type and 2 C-type *LecRLK* genes. Tandem duplication and segmental duplication appear to be the main mechanisms of gene expansion in the soybean *LecRLK* (*GmLecRLK*) gene family. According to our phylogenetic analysis, G-type and L-type *GmLecRLK* genes can be organized into fourteen and eight subfamilies, respectively. The subfamilies within the G-type *GmLecRLKs* differ from each other in gene structure and/or protein domains and motifs, which indicates that the subfamilies have diverged. The evolution of L-type *GmLecRLKs* has been more conservative: most genes retain the same gene structures and nearly the same protein domain and motif architectures. Furthermore, the expression profiles of G-type and L-type *GmLecRLK* genes show evidence of functional redundancy and divergence within each group. Our results contribute to a better understanding of the evolution and function of soybean *LecRLK*s and provide a framework for further functional investigation of them.

## Introduction

Cell surface receptors play important roles in perceiving and processing signals that arrive at the cell. One large family of such cell surface receptors is the receptor-like kinase (RLK) family^1^. RLKs contain three functional domains: an N-terminal extracellular domain, a transmembrane domain and an intracellular kinase domain^2^. The extracellular domains of RLK proteins are highly divergent and usually are comprised of different protein domains, such as a leucine-rich repeat (LRR) domain, and a lectin domain. The kinase domains (KDs), which are fairly conserved, contain 12 conserved subdomains that fold into a three-dimensional catalytic core with a two-lobed structure^3,4^. Based on the structure of the extracellular domains and on a phylogenetic analysis of the kinase domains, RLK proteins of *Arabidopsis thaliana* were classified into more than 15 families^2^.

The lectin receptor-like kinases (LecRLKs) are a class of RLKs that contain a lectin domain within the extracellular domain. Based on the class of lectin domain they contain, LecRLKs have been further classified into three categories, the G-, L-, and C-type lecRLKs^5–7^. The G-type LecRLKs (previously called B-type LecRLKs) contain a bulk-lectin (B-lectin) or a D-mannose binding lectin domain within the N-terminal extracellular domain. G-type LecRLKs are also known as S-domain RLKs due to the presence of an S-locus glycoprotein domain in these proteins and due to their role in self-incompatibility in plants^8–11^. In many G-type LecRLK proteins, the B-lectin domain is also accompanied by an epidermal growth factor (EGF)-like domains and/or a Plasminogen-apple-nematode (PAN) domain^5,7^. The cysteine-rich EGF-like domain^2^ probably takes part in the formation of disulfide bonds, and the PAN motif is believed to be involved in protein-protein and protein-carbohydrate interactions^12–14^. The L-type LecRLKs contain a characteristic legume lectin domain in the extracellular region. This domain resembles soluble legume lectin proteins, which are ubiquitous in leguminous seeds and are involved in binding monosaccharides^15^. The legume lectin domains of LecRLKs are unlikely to be involved in binding monosaccharides; instead, they could interact with complex glycans or with hydrophobic ligands^15^. The C-type LecRLKs contain a calcium-dependent carbohydrate-binding lectin domain in the N-terminal extracellar domain. This domain is commonly found in a large number of mammalian proteins that mediate innate immune responses^16^.

LecRLKs play important roles in plant development and stress responses. They have been found to be involved in seed germination^17^, lateral root development^18^, pollen development^19^, cotton fiber development^20^, legume-rhizobia symbiosis^21,22^, hormone signaling^23,24^, defenses against pathogens and insect pests^25–30^, and responses to abiotic stresses such as salt, drought, wounding, or extreme temperature^7,31,32^.

The rapid increase in the number of sequenced plant genomes has facilitated research into the identity and evolutionary history of whole gene families at a genomic level. For example, We and several research teams have investigated the membership and evolution of the leucine-rich repeat receptor-like protein kinase (LRR-RLK) gene family in plant species for which a complete genome sequence is available, including a moss and a lycophyte^33^, the basal angiosperm *Amborella trichopoda*^34^ and other angiosperm species^35–41^. However, genome-level investigations into the *LecRLK* gene family have only been performed in *Arabidopsis thaliana, Populus trichocarpa*, rice and bread wheat^1,5,42^, while little information is available for other plant species. Soybean (*Glycine max*) is the most important legume used as a protein source for animal feed, and it is an economically important source of vegetable oil for human consumption^38^. Research by Zhou *et al*.^38^ indicated that most gene families have more complex evolutionary histories in soybean than in *Arabidopsis thaliana*, rice, or Poplar. Considering the large number of *LecRLK* genes and their important role in the soybean development and stress responses^21,22^, without clearly understanding of the complex evolutionary histories of them retard the functional studies of soybean *LecRLK* genes.

In this study, we performed a genome-wide search for *LecRLK* gene sequences in soybean and identified a total of 123 G-type, 60 L-type and two C-type putative *LecRLK* genes. We performed a phylogenetic analysis of the G-type and L-type *LecRLK* sequences we identified and classified them into subfamilies. Furthermore, we analyzed the predicted gene structures, and protein domain and motif architectrues of the *LecRLK* sequences to explore the functional evolution of this gene family. Finally, we profiled the expression of the predicted genes. Our results contribute to a better understanding of the evolution and function of soybean *LecRLK* genes and provide a framework for further investigations into the functions of them.

## Results

### Identification and genome-wide distribution of *LecRLK* genes in soybean

In total, we identified 185 non-redundant *LecRLK* sequences in the soybean genome. We further classified the sequences into 123 G-type, 60 L-type and two C-type *GmLecRLKs* on the basis of the presence of an extracellular bulb lectin (PF01453), legume lectin (PF00139), or c-lectin (PF00059), respectively, in each sequence. We calculated the percentage of all protein-coding genes represented by *LecRLK* genes in this species and four other angiosperm species in which *LecRLK* genes have been studied on a genome-wide level. *LecRLK*s account for 0.33% of all genes in soybean, while they account for 0.27% and 0.26% in *A. thaliana* and *T. aestivum* and 0.56% and 0.78%. in *P. trichocarpa* and *O. sativa*, respectively. The percentages of genes accounted for by G-type and L-type *LecRLK*s in these species range from 0.117% to 0.449% and from 0.085% to 0.323%, respectively.

**Figure 1:**
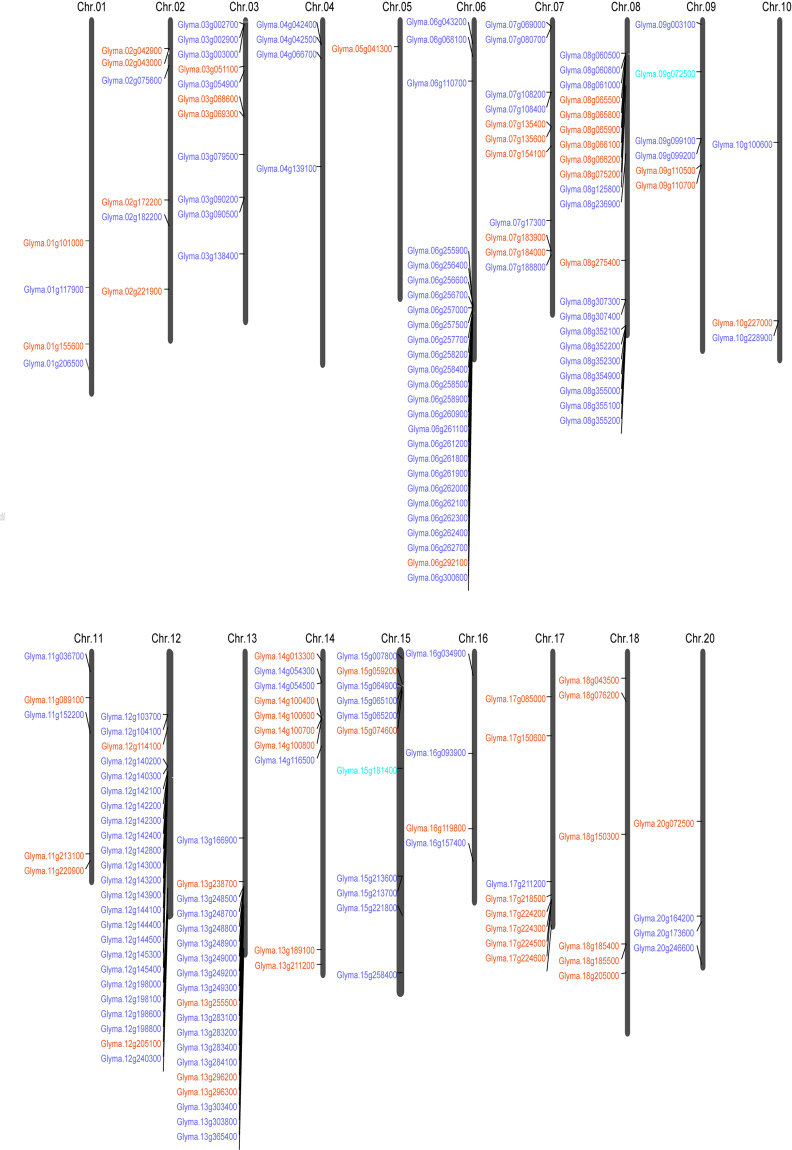
Distribution of *LecRLK* genes on soybean chromosomes. The chromosome numbers are given at the top of each chromosome.

Previous study showed that most soybean genome sequences can be assembled into 20 chromosomes^43^. All 185 *GmLecRLKs* were distributed across 19 soybean chromosomes (Fig. 1), with the exception of one *GmLecRLK* gene that was detected on a scaffold with an indeterminate chromosomal location. Chromosome 4 only contains G-type *GmLecRLKs*, chromosome 18 only contains L-type *GmLecRLKs*, and each of the remaining 17 chromosomes contains both G-type and L-type *GmLecRLKs*. Among these, chromosomes 6, 12 and 13 contain the largest numbers of G-type *GmLecRLKs*, while chromosomes 8, 14, and 17 contain the largest numbers of L-type *GmLecRLK*s. Furthermore, 69.11% (85/123) of the G-type *GmLecRLKs* were found as clusters of tandem repeats. On chromosome 6, there are two nearby clusters with 10 and 11 G-type *GmLecRLK* genes (Fig. 1), respectively. On chromosome 13, there is one cluster with 7 G-type *GmLecRLK* genes. All other G-type clusters contain 2–4 genes. 41.67% (25/60) of L-type *GmLecRLKs* were found as clusters of tandem repeats. We found one cluster with 5 genes on chromosome 8, two clusters with 4 genes each on chromosomes 14 and 17, and several other clusters that each contain 2 genes (Fig. 1).

Based on a comparison with the plant genome duplication database (PGDD), we found that a total of 19 and 17 paralogous gene pairs of G-type and L-type *GmLecRLKs*, respectively, were resulted from segmental duplications (Supplemental Table S1). We calculated the values of Ka/Ks to characterize the selective pressure of these gene pairs. The results showed that the Ka/Ks ratios of all these gene pairs were less than 0.5, suggesting purifying selection of these genes.

### Phylogenetic analysis of *GmLecRLK* genes

**Figure 2:**
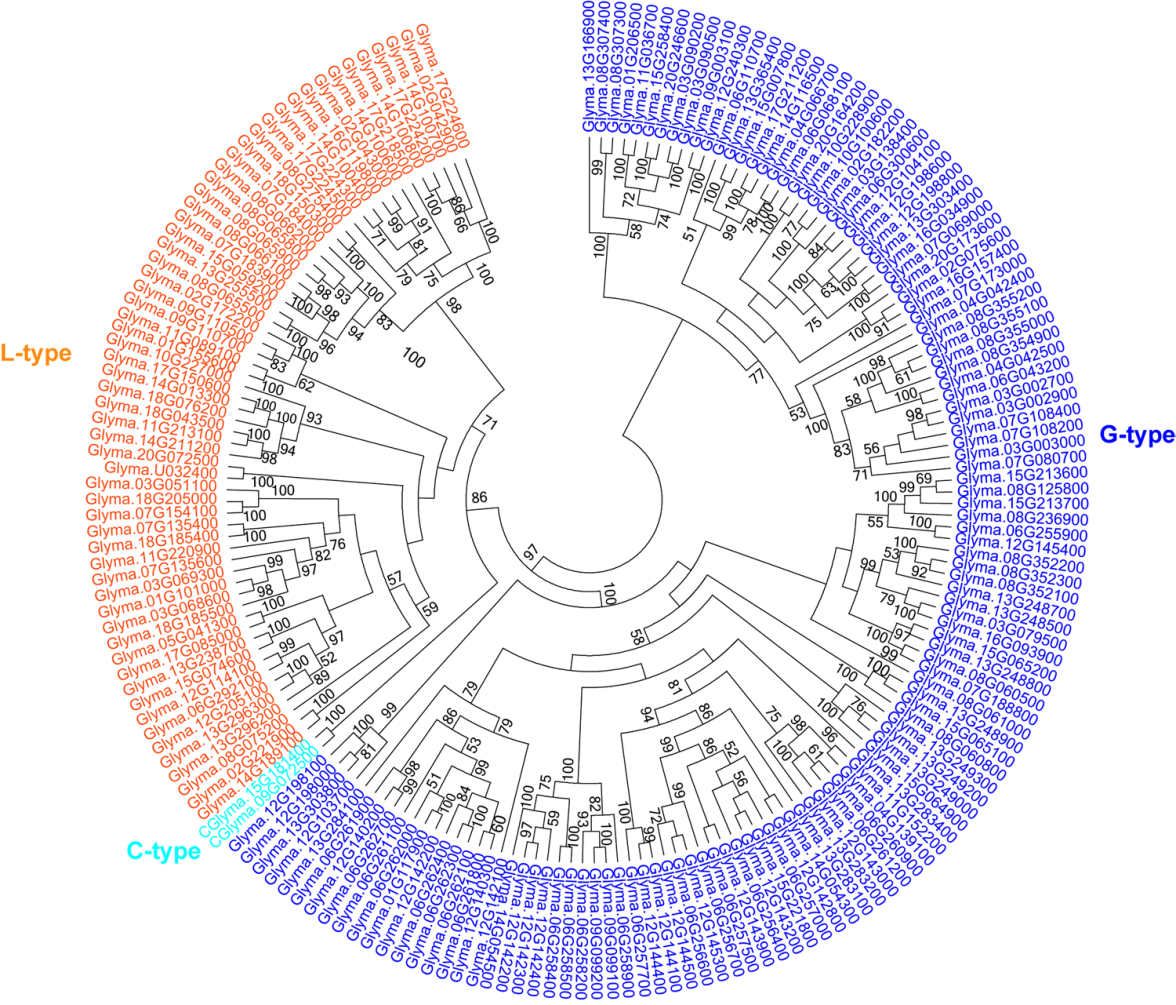
Phylogenetic tree based on all *LecRLK* genes in soybean genome. The phylogenetic tree was constructed using the maximum likelihood method based on amino acid sequences of the kinase domain alone.

To further validate our domain-based classification of *GmLecRLKs*, all *GmLecRLK* genes identified in this study were combined to construct a phylogenetic tree. In the phylogenetic analysis using only the KD sequences, *GmLecRLK* genes clearly separated into three clades (Fig. 2): one clade consisted of 123 G-type *GmLecRLKs*, one consisted of 60 L-type *GmLecRLKs* and one consisted of two C-type *GmLecRLKs*. This result is consistent with the protein architecture-based classification of *GmLecRLK*s.

**Figure 3:**
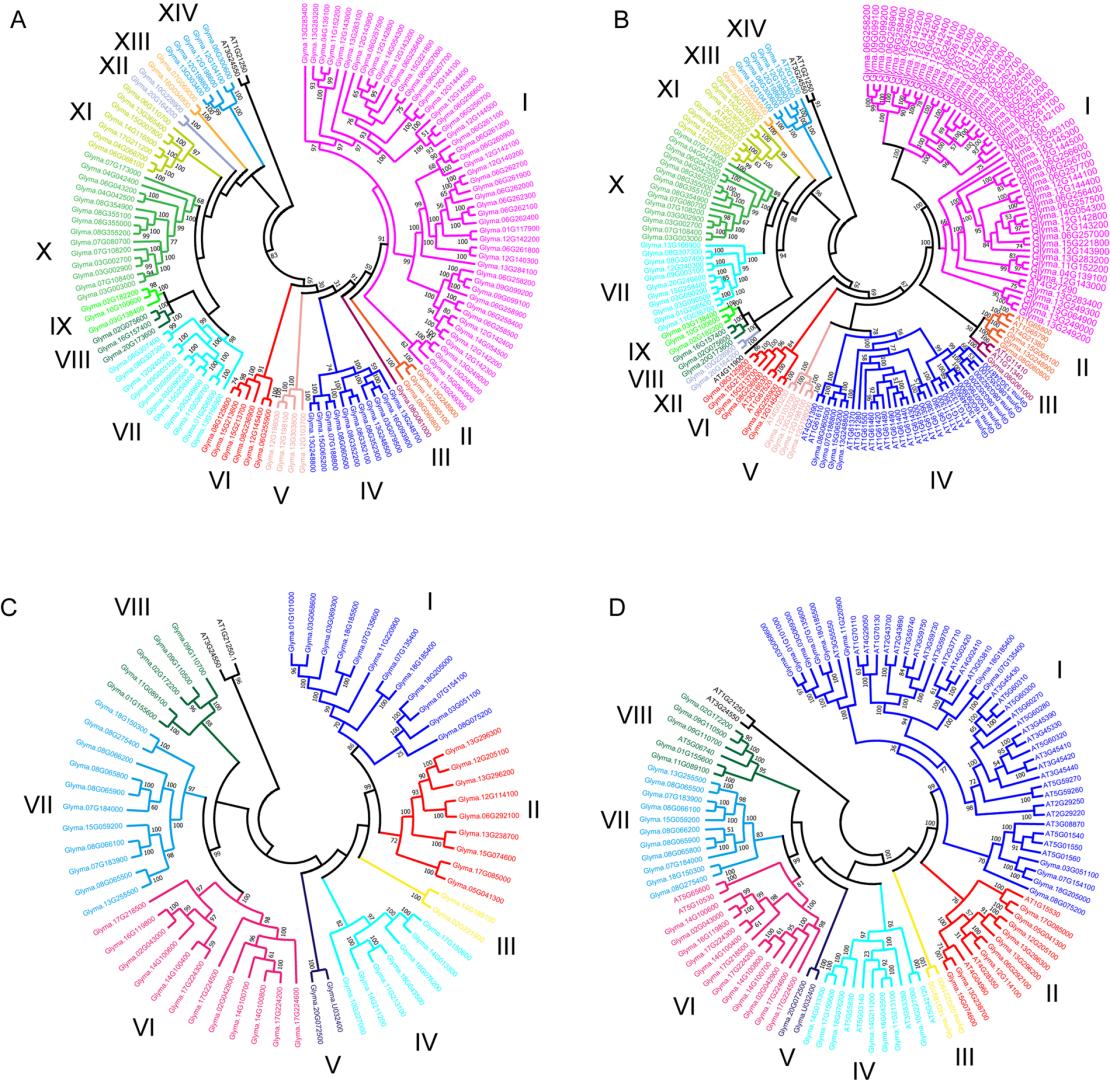
Phylogenetic trees of *LecRLK* gene classes. (**A**) Maximum likelihood tree of G-type *LecRLK* genes in soybean. (**B**) Maximum likelihood tree of G-type *LecRLK* genes in soybean and *Arabidopsis thaliana*. (**C**) Maximum likelihood tree of L-type *LecRLK* genes in soybean. (**D**) Maximum likelihood tree of L-type *LecRLK* genes in soybean and *Arabidopsis thaliana*. Trees were constructed using full-length amino acid sequences. Bootstrap values of major clades are shown around the branches.

To explore the phylogentic relationships within each *GmLecRLK* class, full-length amino acid sequences from each class were analyzed separately. Phylogenetic trees were constructed using maximum likelihood (ML). As shown in the ML tree (Fig. 3A), the 123 G-type sequences clustered into distinct clades, indicating that these natural groups can be assigned to different subfamilies. In total, G-type *LecRLKs* in soybean were classified into 14 subfamilies. All subfamilies were supported as clades with high bootstrap support. This phylogenetic analysis also provided some information about the evolutionary relationships among the subfamilies within the G-type *GmLecRLKs*. For example, the ML tree showed that subfamily I and subfamily II were sister clades and that the clade containing those two subfamilies was the sister of subfamily III. To further explore the phylogenetic relationships among soybean G-type LecRLK proteins and Arabidopsis G-type LecRLK proteins, we performed a phylogenetic analysis of these sequences in the two species. As shown in Fig. 3B, in this analysis, the sequences from *Arabidopsis* appeared in 8 subfamilies defined according to the GmLecRLK phylogenetic analysis.

Using the same process, we subjected 60 L-type GmLecRLK sequences to phylogenetic analysis. The ML tree (Fig. 3C) showed eight major clade (I to VIII) with high bootstrap support. Similarly, L-type *LecRLKs* in soybean were classified into 8 subfamilies. Within the tree, groups I and II are sister groups, and groups VI and VII are sister groups. We also generated an ML tree (Fig. 3D) based on the full-length amino acid sequences of L-type GmLecRLKs and AtLecRLKs. On this tree (Fig. 3D), GmLecRLK sequences from each subfamily of Fig. 3C also included in one clade, respectively. Therefore, we adopted the soybean *LecRLK* subfamily nomenclature in Fig. 3C to label corresponding subfamilies in Fig. 3D. In total, *LecRLKs* from soybean and *Arabidopsis* fell into eight subfamilies: three clades (III, V, VII) contained no *Arabidopsis* L-type *LecRLKs*, and five clades (I, II, IV, VI and VIII) contained at least one *Arabidopsis* L-type *LecRLK*. In previous study^6^, all *Arabidopsis* L-type *LecRLKs* were clustered into 9 clades and 5 singletons (we named these clades and singleton sequence from right to left of Fig. 2a, group 1 to 9, S1 to S5 for convenience). In the present study (Fig. 3D), subfamily I included all *Arabidopsis LecRLK* sequences from group 1 to 6, and S1 in that study, subfamily II included all *Arabidopsis LecRLKs* from group 7 and S2, subfamily IV contained all *Arabidopsis LecRLKs* from group 8, S3 and S4, subfamily VI contained all Arabidopsis *LecRLKs* from group 9, and subfamily VIII contained all *Arabidopsis LecRLKs* from S5.

In addition, we estimated the distance of *lecRLK* genes by MEGA6^44^. The results (Supplementary Table S2) showed that sequences in the same subfamily or cluster are more similar, consistent with the phylogenetic relationships.

### *GmLecRLK* gene structures

**Figure 4:**
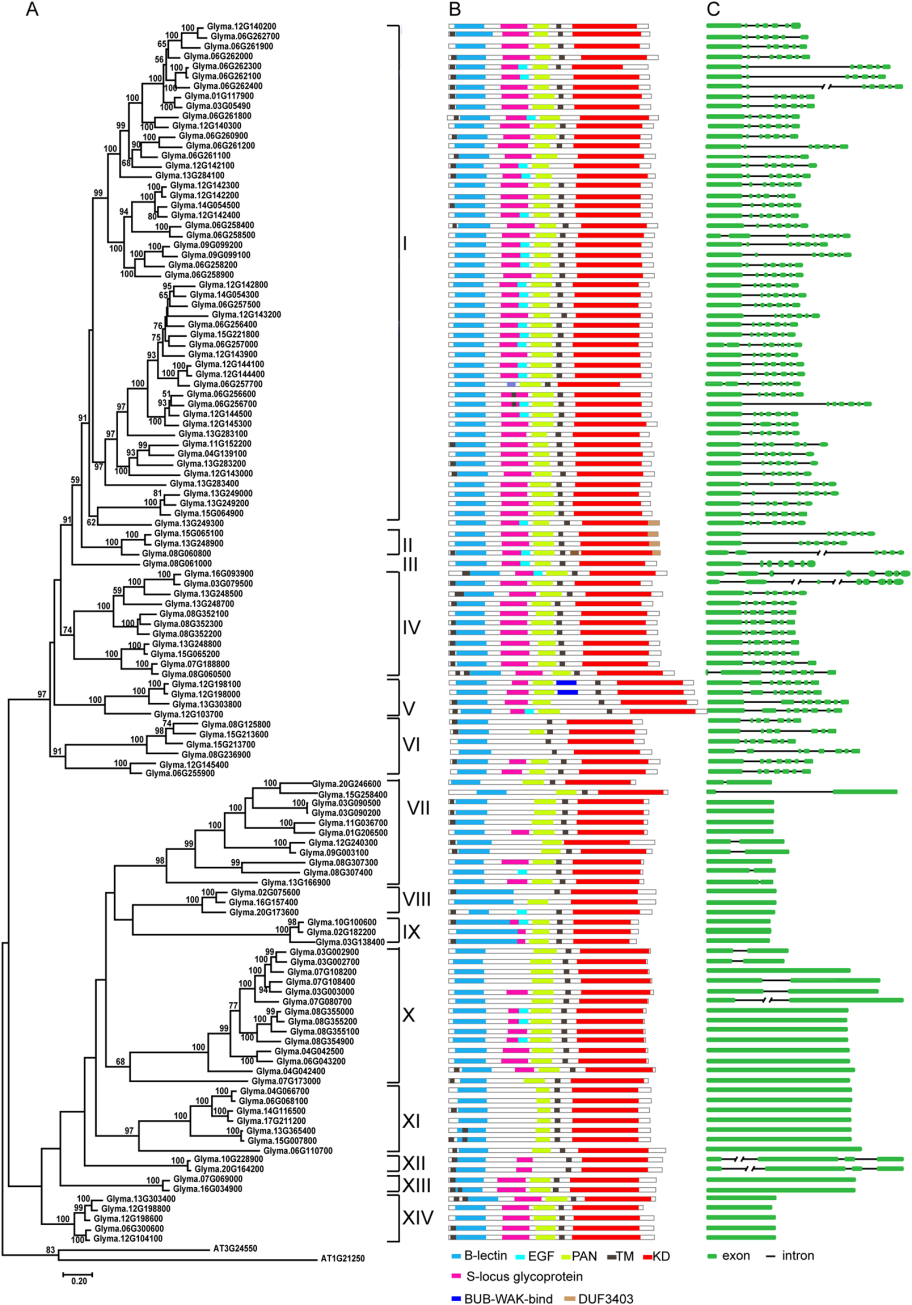
ML tree of G-type *LecRLK* genes from soybean, with corresponding protein structures, and gene structures. (**A**) ML tree of 123 G-type LecRLK proteins from soybean. The subfamily names are shown on the right. (**B**) Protein structures of G-type LecRLK proteins. (**C**) Gene structures of G-type LecRLK proteins. The green boxes represent exons, the lines represent introns, and each line with double slash indicates a long intron.

**Figure 5:**
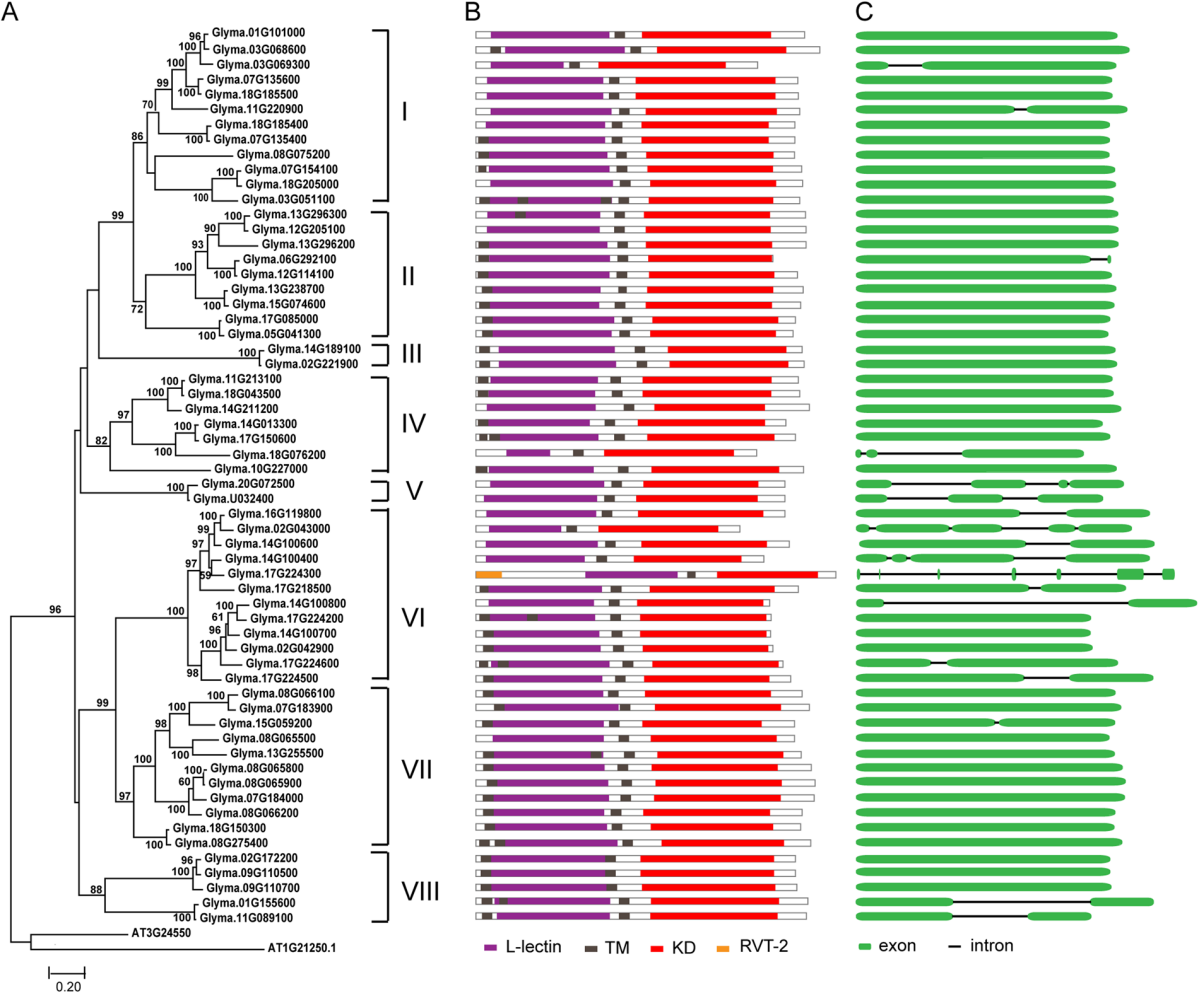
ML tree of L-type *LecRLK* genes from soybean, with corresponding protein structures, and gene structures. (**A**) ML tree of 60 L-type LecRLK proteins from soybean. The subfamily names are shown on the right. (**B**) Protein structures of L-type LecRLK proteins. (**C**) Gene structures of L-type LecRLK proteins.

**Figure 6:**
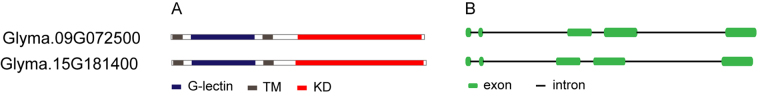
Protein structures and gene structures of C-type *LecRLK* genes from soybean. (**A**) Protein structures of C-type LecRLK proteins. (**B**) Gene structures of L-type LecRLK proteins.

We analyzed the gene structures of the 123 G-type and 60 L-type *GmLecRLK* genes we identified, and we mapped them in the phylogenetic trees (Figs 4C and 5C). As shown in Fig. 4C, we found that most of the closely related G-type *GmLecRLKs* have roughly the same number and position of introns, strongly supporting their close evolutionary relationships. Almost all members of subfamilies I, II, III, IV and VI have six introns in their coding sequences, all members of group V have seven introns, all members of subfamily XII have two introns, while most members of other subfamilies have no introns. Comparative gene structure analysis of 60 L-type *GmLecRLK* gene sequences revealed that members of subfamily V have two or three introns in their coding sequences, while most members of all other groups have no introns (Fig. 5C). We also analyzed the gene structures of two C-type *GmLecRLK* genes, the result showed they both have four introns in their coding sequences (Fig. 6B).

### Protein domain and motif analyses

Based on the results of our CDD and ScanProsite analyses, the predicted domain architecture of each LecRLK protein was mapped; domain maps are shown to the right of the phylogenetic trees in Figs 4 and 5. We identified the B-lectin, L-lectin, and C-lectin domains only in G-type, L-type, and C-type GmLecRLK*s*, respectively, while the kinase domain occurs in all GmLecRLKs. With the exception of one predicted protein that contains an RVT_2 superfamily domain, all L-type GmLecRLKs only contain an L-lectin and a kinase domain (Fig. 5B). Similarly, the two C-type GmLecRLKs only contain an C-lectin and a kinase domain (Fig. 6A). We observed that the G-type GmLecRLKs have more diverse domain architectures than the other two types (Fig. 4B). In addition to their lectin and kinase domains, many G-type GmLecRLKs contain an S-locus glycoprotein domain, an EGF domain and a PAN/apple domain. We called B-lectin, kinase, S-locus glycoprotein, and PAN/apple domains the four basic domains. We found that although some subfamilies have the same domain compositions, many subfamilies of G-type GmLecRLKs are usually characterized by different typical compositions of these domains. For example, all members of subfamilies IV, V, IX, XIII and XIV contain the four basic domains, 41% (21/51) of subfamily I members and all of subfamily III members contain four basic domains and an EGF domain, all members of subfamily II contain four basic domains and a DUF3403 domain, most members of subfamilies VI, VII and XI do not contain a S-locus glycoprotein domain, and members of subfamily VIII only contain B-lectin and kinase domains.

We identified the conserved motifs in GmLecRLK amino acid sequences using the MEME program. The MEME analysis showed that G-type GmLecRLKs have more diverse motif architectures than do L-type GmLecRLKs (Fig. 4). We identified 15 motifs in GmLecRLKs, which we label M1 to M15 from the N- to the C-terminus (Supplemental Table S2). In the G-type GmLecRLKs, M1 to M4 correspond to the B-lectin domain, M5 corresponds to the EGF domain, M6 corresponds to the PAN domain, and M8 to M15 correspond to the KD. The G-type subfamilies I, II, III and IV contain motifs M1 to M15, but the other subfamilies are missing one or more of these motifs. For example, subfamilies V and VI do not contain motifs M5 and M6, subfamilies VII and XII do not contain motifs M5 and M14, and subfamilies X, XI, XIII, XIV do not contain motif M14, (Supplemental Figure S1). In the L-type GmLecRLKs, motifs M1 through M7 correspond to the B-lectin domain, and M8 through M15 correspond to the KD. All subfamilies of L-type GmLecRLKs contain motifs M1 through M15, with the exception of subfamily III, which lacks motifs M12 and M13, and subfamily V, which lacks motifs M2 and M7 (Supplemental Table S2). We did not perform MEME analysis on C-type GmLecRLKs since there are only two sequences.

### Transcriptional profile analysis of *GmLecRLK* genes

Little is known about the functions of *LecRLKs* in soybean. As a first attempt to provide insights into their potential functions, we used RNA-seq data from the Phytozome v10 database to profile the relative expression of *GmLecRLK* genes across various tissues (Fig. 6). We observed that more of the G-type genes were expressed in higher quantity in leaves and roots (Fig. 6A). Further, some G-type *GmLecRLK* genes had similar expression patterns to others in the same subfamily, suggesting the functional redundancy of genes within a cluster. Conversely, some genes had different expression patterns from others in the same cluster, suggesting functional divergence within a subfamily. For example, in subfamily I, eight genes showed similar, high expression levels only in leaves, whereas nine genes showed high expression levels only in roots (Fig. 6A). In subfamily IV, two, four, one, and one genes, respectively, were expressed at high levels in leaves, roots, flowers, and seeds, and four genes were expressed at high levels in two tissues. Contrasting with the expression patterns of G-type genes, we observed that more of the L-type genes were expressed in high quantity in leaves and seed (Fig. 6B). Similarly, we observed both similar and divergent expression patterns among L-type *GmLecRLK* genes within the same subfamily. For example, in subfamily I (Fig. 6B), four and two genes, respectively, were highly expressed in leaves and seeds, and one each was highly expressed in roots, flowers, root hairs and pods. In subfamily VI, three, one and one genes, respectively, were highly expressed in leaves, seeds and roots, while eight genes were highly expressed in two or more tissues. The two C-type *GmLecRLK* genes showed expression in all tissues, with the highest expression levels in SAM (Fig. 6C).

### Quantitative real time RT-PCR analysis

**Figure 7:**
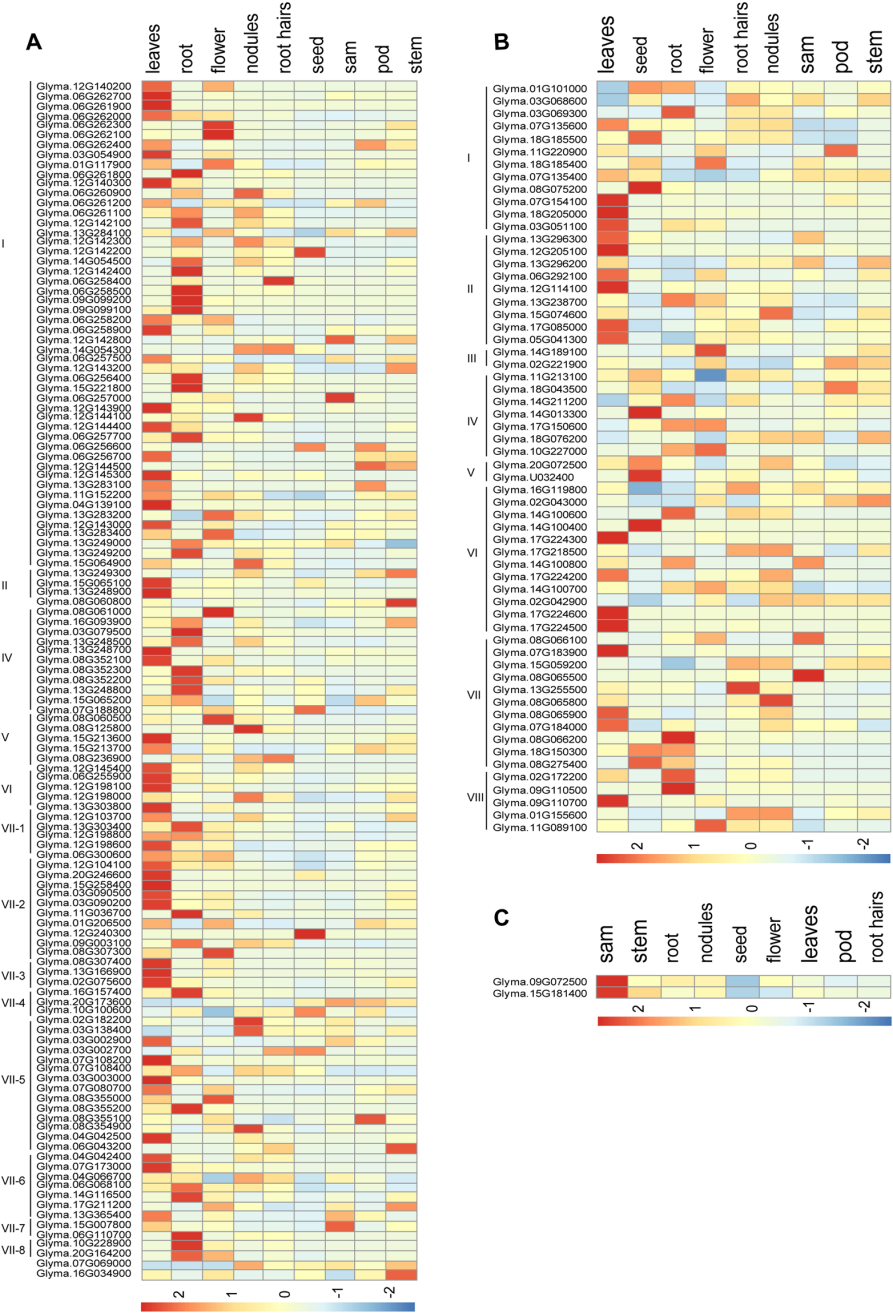
*LecRLK* gene transcript abundance in soybean. Transcript abundance of G-type, L-type and C-type genes are shown in (**A**), (**B**) and (**C**), respectively. The color scale represents the expression values.

**Figure 8:**
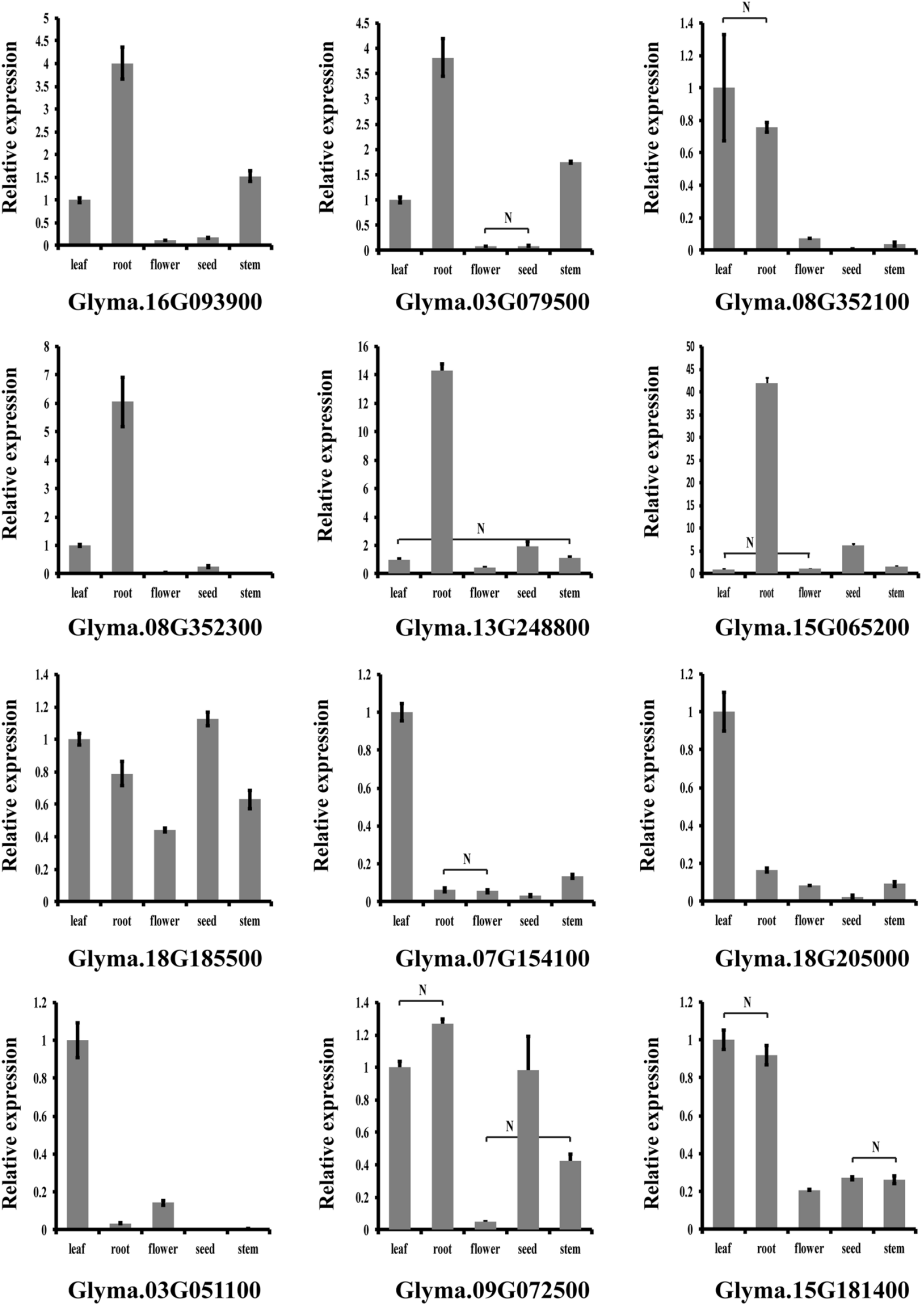
Transcription level of 12 LecRLK genes in various tissues as analyzed by qRT-PCR. Gene expression levels were normalized with ACT11 transcript values. Statistical analysis was conducted by t-test to determine the significance of the relative expression of individual genes among different tissues. Since there were significant differences in the expression level for each gene in most pairs of tissues (*P* < 0.05, t-test), only pairs of tissues between which there was no significant difference in expression were marked by N.

In order to validate the expression patterns of *GmLecRLK* genes, the expression levels of 10 randomly selected genes from subfamily IV from G-type and subfamily I from L-type *GmLecRLK*s and 2 genes of C-type *GmLecRLK*s were detected by using Quantitative Real-Time PCR (qRT-PCR) across five tissues. The results showed that most genes exhibited significant differences (*P* < 0.05, t-test) in expression among different tissues (Fig. 8). The results also showed that expression patterns identified by RNA-seq analysis were consistent with that identified by qRT-PCR. For example, both the RNA-seq data and qRT-PCR analysis showed that five genes (Glyma.16G093900, Glyma.03G079500, Glyma.08G352300, Glyma.13G248800 and Glyma.15G065200) from subfamily IV of G-type *GmLecRLKs* were mainly expressed in root (Figs 7A and 8). Similarly, both the RNA-sequencing and qRT-PCR analysis showed that three genes (Glyma.07G154100, Glyma.18G205000 and Glyma.03G051100) from subfamily I of L-type *GmLecRLK*s were mainly expressed in leaves (Figs 7A and 8).

### Co-expression analysis

To study the expression divergence between family members of G-type, L-type and C-type *GmLecRLKs*, we performed co-expression analysis of gene pairs across various tissues. As a result, we detected 57 co-expressed gene pairs (adjusted P < 0.01) in G-type *GmLecRLK* genes (Supplementary Table S3), representing 37 genes. However, we did not detect any co-expressed genes in other types of *GmLecRLK* genes. These results suggested there are divergence in the promoter regions of *GmLecRLK* genes.

## Discussion

In this study, we identified 185 *LecRLK* genes in the genome of soybean. On the basis of identity of lectin domains, 185 *LecRLKs* were classified into 123 G-type, 60 L-type and 2 C-type *LecRLKs*. Our phylogenetic analysis based on the kinase domain sequences of all *GmLecRLK* genes showed that the three types of *GmLecRLK* genes were clearly separated into three different clades (Fig. 2), which further supports the classification of *GmLecRLKs* into three types. Previous studies have identified *LecRLK* genes in some species by analyzing genome sequences. For example, in the genomes of *A. thaliana*, *P*. *trichocarpa*, *O. sativa* and *T. aestivum*, 75, 231, 173, and 263 *LecRLK* genes, respectively, have been identified^1,5,42^. The number of *LecRLK* genes in soybean and rice is about 2.5 times that in *A. thaliana*, the number of *LecRLK* genes in *P. trichocarpa* is about 3 times that in *A. thaliana*, and the number of *LecRLK* genes in *T. aestivum* is about 3.5 times that in *A. thaliana*. Hence, the copy numbers of *LecRLK* genes among angiosperm species are quite diverse. Differences in the copy numbers of *LecRLK* genes may be due to differential expansion rates of *LecRLK* genes in different genomes, but it may also be due to differences in genome size. To distinguish these factors, we compared the proportions of *LecRLK* genes among all protein-coding genes in different genomes. We observed that *LecRLK* genes represent a similar percentage of all genes in the *G. max*, *A*. *thaliana*, and *T. aestivum* genomes (Table 1); therefore, the copy number differences among these species may be due to the differences in genome size. However, the percentages of *LecRLK* genes among all *P. trichocarpa* and *O. sativa* genes are 2–2.8 times the percentages in *A. thaliana* and *G. max* (Table 1). This suggests that the differences in the copy number of *LecRLK* genes in these genomes may be due to differential expansion rates of *LecRLK* genes. We previously reported similar results for the *LRR-RLK* gene family^34^. We also found that expansion rates differ between G-type and L-type *LecRLK* genes and range from 0.117% to 0.449% for G-type and from 0.085% to 0.323% for L-type genes. When we compared the expansion rate of G-type and L-type *LecRLK* genes in each genome, we found that in soybean, *P. trichocarpa*, *O. sativa* and *T. aestivum*, the G-type *LecRLK*s were expanded to a greater extent than L-type *LecRLK*s. This contrasts with the results of a previous study, which indicated that L-type *LecRLK*s were expanded to a greater extent than G-type *LecRLK*s in *Arabidopsis*^34^.

**Table 1:** Percentages of all protein-coding genes accounted for by *LecRLK* genes.

Plant species	Number of protein-coding genes	All *LecRLK*s	G-type	L-type	C-type
*A. thaliana*	27,416	75 (0.27)	32 (0.117)	42 (0.153)	1
*P*. *trichocarpa*	41,335	231 (0.56)	180 (0.435)	50 (0.121)	1
*G. max*	56,044	185 (0.33)	123 (0.219)	60 (0.107)	2
*O. sativa*	22,273	173 (0.78)	100 (0.449)	72 (0.323)	1
*T. aestivum*	99,386	263 (0.26)	177 (0.178)	84 (0.085)	2

There are several mechanisms by which genes are duplicated, chiefly tandem duplication, segmental duplication (genome duplication), and transpositional duplication^45^. Previous studies have demonstrated that tandem duplication and segmental duplication/genome duplication played a major role in the expansion of LecRLKs in some species, such as *Brassica* species, *P. trichocarpa*, and *T. aestivum*^1,42,46^. In our study, 69.11% (85/123) of the G-type *GmLecRLK*s and 41.67% (25/60) of the L-type *GmLecRLK*s were found as clusters of tandem repeats. We also found some super tandem replicate gene clusters, which have also been reported in *P. trichocarpa*^1^. For example, on chromosome 6, there are two nearby clusters with 10 and 11 G-type *GmLecRLK* genes, respectively (Fig. 1). Further, using the PGDD, we found that 62 G-type and 35 L-type *GmLecRLK* genes were located on the retention regions after segmental/genome duplication (Supplemental Table S1). Hence, tandem duplication and segmental duplication might be the main mechanisms of gene expansion in the soybean *LecRLK* gene family.

After a duplication event, duplicated genes often accumulate mutations, which can lead to their functional divergence^47^. Our phylogenetic analysis showed that both G-type and L-type *GmLecRLK* genes were clustered into different clades, suggesting that *GmLecRLK* genes have diverged over time. Furthermore, gene structure analysis and protein domain and motif analysis demonstrated divergence within both the G-type and the L-type *GmLecRLK* gene clades. For example, there are four main gene structure groups among G-type *GmLecRLK* genes, characterized by the presence of six introns, seven introns, three introns and no introns in the coding region, respectively (Fig. 4C). Within the group with six introns (almost all members of subfamilies I, II, III, IV and VI), the domain compositions differ among the subfamilies. Subfamilies I and III contain five domains: four basic domains (B-lectin, kinase, S-locus glycoprotein, PAN/apple) and EGF. Subfamily II contains the four basic domains and a DUF domain. Subfamily IV only contains the four basic domains. Most members of subfamily VI do not contain the glycoprotein domain. Similarly, although members of subfamily VII to XI, XIII and XIV have the same gene structure, different subfamilies have different domain compositions (Fig. 4B). For example, subfamilies IX, XIII and XIV contain the four basic domains mentioned above, while subfamily VII contains B-lectin, kinase, and PAN domains. Most members of subfamily VIII only contain the B-lectin and kinase domains. The subfamilies X and XI do not contain the glycoprotein domain. The MEME motif analysis can also clarify some subfamilies, for example, subfamily VI did not have motif M5, subfamilies VII and XII did not have motif M5 and M14; subfamily XIII and XIV did not have motif M14. Previous studies have demonstrated that introns (gene structure) have important roles in cellular and developmental processes via alternate splicing or gene expression regulation^48^, and that different domains such as B-lectin, kinase, S-locus glycoprotein, PAN/apple and EGF domain have different functions. Hence, each subfamily of G-type *GmLecRLK* genes differs from the others either in the gene structure or protein domains, or motifs, suggesting that the subfamilies have diverged. In our previous study, we investigated the evolution of another *RLK* subfamily, the *LRR-RLK* family^33,34^. Our results suggested that the *LRR-RLK* subfamilies are more divergent than those of the G-type *LecRLK* genes.

On the contrary, the L-type *GmLecRLK* genes are more conserved than the G-type genes. A majority of L-type *GmLecRLK* genes have no intron in the coding region (Fig. 5C). Additionally, our results suggest that the domain composition is conserved among L-type *GmLecRLK* genes. All but one of the L-type GmLecRLKs contain only L-lectin and kinase domains (Fig. 5B). Using MEME analysis, we showed that all subfamilies of L-type GmLecRLK contain the same 15 motifs and arrangement, except that subfamilies III and V each lack two of the motifs. Hence, L-type *GmLecRLKs* are less divergent in their gene structure, protein domain structure, and motifs.

Tissue-specific transcript abundance is often suggestive of a gene’s biological function. Gene expression patterns might therefore offer insights into the potential functions of GmLecRLKs. Our expression analysis showed that some G-type *GmLecRLK* genes of the same subfamily had a similar expression pattern, suggesting possible functional redundancy of genes within a cluster. For example, both the RNA-sequencing and qRT-PCR analysis showed that five genes (Glyma.16G093900, Glyma.03G079500, Glyma.08G352300, Glyma.13G248800 and Glyma.15G065200) from subfamily IV of G-type GmLecRLK genes had the similar expression pattern: they were mainly expressed in roots. The similar expression pattern suggested that they may have redundant function. In the phylogenetic tree, three of these members were clustered with a clade containing AT1g11300/EGM1, suggesting that they may share the same function. EGM1 involve in signaling of mannitol-associated stress response^49^. Similarly, both the RNA-sequencing and qRT-PCR analysis showed that three genes (Glyma.07G154100, Glyma.18G205000 and Glyma.03G051100) from subfamily I of L-type *GmLecRLKs* had the similar expression pattern: they were mainly expressed in leaves (Figs 7A and 8). The similar expression pattern also suggested that they may have redundant function. In the phylogenetic tree, these three genes grouped together with members of *Arabidopsis* A4 subfamily of lectin receptor-like kinases (*At5g01540/lecRKA4.1*, At *5g01550/lecRKA4.2*, *At5g01560/lecRKA4.3*). These proteins have a redundant function in the negative regulation abscisic acid response in seed germination^50^. Conversely, some genes had different expression patterns from others in the same cluster, suggesting functional divergence within a subfamily. For example, one gene (Glyma.18g185500) showed a different expression pattern from that of three genes mentioned above from the same subfamily I of L-type *GmLecRLKs*, and it was more or less uniformly expressed through all tissues or organs (Fig. 8). The different expression patterns suggested these genes may have different function. In contrast with the tissue-specific expression of most G- and L-type genes, the two C-type *GmLecRLK* genes were expressed in all tissues. The co-expression analysis showed that there are 57 co-expression gene pairs (representing 37 genes) in G-type *LecRLKs* and no co-expression gene pairs in G-type and C-type *LecRLKs* (Supplementary Table S3), consistent with the expression data of *LecRLK* genes.

Taken together, our evolutionary, structural and expression analysis suggested divergence of soybean *LecRLK* subfamilies and functional redundancy of the members in the same subfamily. The result of this study shed light on the evolution and function of soybean *LecRLKs*, and provide a framework for further functional investigation of these genes.

## Methods

### Identification of *LecRLK* sequences in the soybean genome

The proteomic sequences of completely sequenced *Glycine Max* genome were downloaded from Phytozome v11.0 (https://phytozome.jgi.doe.gov/pz/portal.html#)^51^. Hidden Markov Model (HMM) profiles (PF00069, PF01453, PF00139, PF00059), which correspond to kinase, B-lectin, L-lectin and C-lectin domains, respectively, were downloaded from pfam (http://pfam.xfam.org/). We retrieved genes containing a kinase domain (KD) by running the hmmsearch program (HMMER 2.3.2) to search the kinase profile (PF00069) against the soybean genomes. Within this set of hypothetical kinase proteins, we then searched for B-lectin, L-lectin and C-lectin HMM profiles (PF01453, PF00139, PF00059) (E value cut-off < 1). Sequences in which we identified a protein kinase domain (PF00069), along with either a B-lectin (PF01453), an L-lectin (PF00139), or a C-lectin domain (PF00059), were considered putative soybean LecRLKs (GmLecRLKs). Identical and defective sequences were identified and eliminated by manual inspection in BioEdit^52^. The candidates were analyzed with TMHMM v. 2.0 (http://www.cbs.dtu.dk/services/TMHMM/)^53^ to confirm the presence of predicted transmembrane domains (TMs). Only sequences that contained a lectin domain within the extracellular domain, a TM, and a KD were considered putative LecRLKs.

We next compared the ratios of *LecRLK* genes to the total number of protein coding genes in several plant genomes, as in our previous study^34^. The numbers of putative *LecRLK*s in the genomes of several angiosperm species were obtained from published papers^1,5,42^. The total numbers of protein-coding genes in each genome were obtained from Phytozome v11.0^51^.

### Analysis of genomic distribution and duplications of *LecRLK* sequences

All putative *GmLecRLK*s identified in this study were mapped onto their corresponding chromosomes. First, the physical positions of the putative genes and the chromosome lengths of each soybean chromosome were obtained from the Phytozome database. Then, an image of the chromosomal location of each *GmLecRLK* gene was generated using MapInspect software (http://mapinspect.software.informer.com/). As in previous literature, a tandem duplication cluster was defined as a region containing two or more genes within 200 kb^34,36,38^. Furthermore, genes within a tandem duplication cluster should show a close relationship in a phylogenetic tree. The segmental duplicated *GmLecRLK* genes were characterized according to the plant genome duplication database (PGDD) (http://chibba.agtec.uga.edu/duplication/). The list of genes in duplicated genomic regions and Ka/Ks values of each gene pairs were retrieved from PGDD. The ratio of Ka and Ks (Ka/Ks) was estimated to characterize the selective pressure, with Ka/Ks = 1, < 1 and > 1, which indicate neutral evolution, purifying selection and positive selection, respectively.

### Amino acid sequence alignment and phylogenetic analysis

Our phylogenetic analyses were performed at two levels. First, to further validate the classification of the putative genes into G-, L-, and C-type *LecRLK*s, all *GmLecRLK* genes identified in this study were combined to construct the phylogenetic trees. Since the N-terminal domains differ among the three *LecRLK* types, and alignments of this region were ambiguous, only the amino acid sequences of the common kinase domain were subjected to phylogenetic analysis. Second, to investigate the phylogenetic relationships among *GmLecRLKs* of the same type, the complete amino acid sequences of L-type and G-type *GmLecRLKs* were, respectively, subjected to phylogenetic analysis. We did not perform a phylogenetic analysis of C-type *GmLecRLKs* since we only found two genes of this type. Next, to explore the phylogenetic relationships among the *GmLecRLKs* we identified and *A. thaliana LecRLKs* (*AtLecRLKs*) reported in a previous study^5^, we combined and performed phylogenetic analyses on the full-length amino acid sequences of G-type *LecRLKs* and L-type *LecRLKs*, respectively, from both species. Arabidopsis receptor-like kinases WAK1 (AT1G21250) and PERK1(AT3G24550) were defined as outgroups, similarly as in previous studies^1,5,6^. Multiple sequence alignments were performed using MAFFT with default settings^54^, after which alignments were manually adjusted in BioEdit^52^. Phylogenetic trees were constructed using the maximum likelihood (ML) method implemented in RAxML^55^. The best-fit amino acid substitution models (LG + G for both datasets) for ML analyses were selected by MEGA6^44^. The starting tree was obtained using BioNJ. Parameter values were estimated from the data. Branch support was estimated from 1000 bootstrap replicates. The trees were rooted at the midpoint.

### Gene structure analysis

Genomic sequences of the *G. max* v.1.0 annotation were downloaded from Phytozome v11.0^51^, after which untranslated regions were removed. Coding sequences were also downloaded from Phytozome v11.0^51^. The gene structures of *GmLecRLKs* were determined by comparing coding sequences with their corresponding genomic DNA sequences, after which these structures were displayed using the Gene Structure Display Server (GSDS) v. 2.0 (http://gsds.cbi.pku.edu.cn/)^56^.

### Protein structure analysis

To predict protein functional domains, the full-length amino acid sequences of GmLecRLKs were subjected to protein domain analyses in the Conserved Domains Database (CDD) (http://www.ncbi.nlm.nih.gov/Structure/cdd/wrpsb.cgi)^57^ and using the ScanProsite tool (http://prosite.expasy.org/scanprosite/)^58^. We used TMHMM v. 2.0 (http://www.cbs.dtu.dk/services/TMHMM/)^52^ to predict transmembrane domains (TMs). Since some motifs, including the EGF-like motifs, were not predicted in CDD, we merged the annotation results to generate a protein domain structure containing all predicted protein functional domains. These protein structures were mapped to each protein in the phylogenetic tree. To further understand the potential functions of the LecRLKs in soybean, we used Multiple Expectation Maximization for Motif Elicitation (MEME) v.4.10.2. (http://meme-suite.org/tools/meme)^59^ to predict all putative motifs in these proteins. MEME was executed in zoop (zero or one occurrence per sequence) mode. Parameters were set as follows: maximum number of motifs, 15; minimum and maximum motif width, 6 and 50, respectively; and default settings for all other parameters.

### Transcriptional profile analysis

For *GmLecRLK* gene expression analysis, RNA-seq data from soybean roots, root hairs, nodules, leaves, stems, flowers, shoot apical meristems (SAM), pods, and seeds were obtained from Phytozome v10. We generated a heat map of the *GmLecRLK* genes using the pheatmap package in R (https://www.r-project.org/).

### Quantitative real time RT-PCR analysis

Soybean plants were grown on soil for two months with a day length of 16 h at 25. Root, stems, leaves, flowers and seeds were collected for total RNA extraction. Total RNA was isolated using the Plant RNA Kit (Magen, China) . One microgram of total RNA was used to synthesize cDNA using FastQuant RT Kit (Tiangen, China). Quantitative Real-Time PCRs (qRT-PCR) were carried out using SYBR Green Master Mix Reagent (Takara, Japan) according to the manufacturer’s protocol. Sequences of primers used were shown in Supplemental Table S4. Reactions were performed on a ABI7500 (ABI, USA). The following thermal cycle conditions were used: 95 for 20 s and 58 for 20 s; 72 for 30 s. All reactions were performed in triplicated from three independent pooled samples. Relative quantification of each gene, corresponding to the expression level of *ACT11*, was analyzed using 2^−ΔΔct^ method^60^. Student’s t test (P < 0.05) was used to determine the significance of the relative expression of individual genes among different samples.

### Co-expression analysis

Co-expression between gene pairs is determined by computing the pearson correlation of expression profiles of different type soybean LecRLKs gene pairs across tissues. We choose to use a Pearson correlation adjusted P value of 0.01 as a threshold (bonferroni corrected). We used this algorithm to study the expression similarity between the gene pairs among the families.

## Electronic supplementary material

Dataset 1

Dataset 2

Dataset 3

Dataset 4

Supplementary Information

## References

[1] Yang, Y. I. *et al*. Genome-wide analysis of lectin receptor-like kinases in *Populus*. *BMC Genomics* **17**10.1186/s12864-016-3026-2 (2016). 10.1186/s12864-016-3026-2 PMC5007699 27580945

[2] Shiu SH, Bleecker AB (2001). Receptor-like kinases from *Arabidopsis* form a monophyletic gene family related to animal receptor kinases. Proc. Natl. Acad. Sci. USA.

[3] Hanks SK, Quinn AM, Hunter T (1988). The protein kinase family: conserved features and deduced phylogeny of the catalytic domains. Science.

[4] Hanks SK, Hunter T (1995). Protein kinases 6. The eukaryotic protein kinase superfamily: kinase (catalytic) domain structure and classification. FASEB J..

[5] Vaid N, Pandey PK, Tuteja N (2012). Genome-wide analysis of lectin receptor-like kinase family from *Arabidopsis* and rice. Plant Mol. Biol..

[6] Bouwmeester K, Govers F (2009). *Arabidopsis* L-type lectin receptor kinases: phylogeny, classification, and expression profiles. J. Exp. Bot..

[7] Vaid N, Macovei A, Tuteja N (2013). Knights in Action: Lectin Receptor-Like Kinases in Plant Development and Stress Responses. Mol. Plant.

[8] Takasaki T (2000). The S receptor kinase determines self-incompatibility in *Brassica* stigma. Nature.

[9] Kachroo A, Schopfer CR, Nasrallah ME, Nasrallah JB (2001). Allele-specific receptor-ligand interactions in *Brassica* self-incompatibility. Science.

[10] Stein JC, Howlett B, Boyes DC, Nasrallah ME, Nasrallah JB (1991). Molecular cloning of a putative receptor protein kinase gene encoded at the self-incompatibility locus of *Brassica oleracea*. Proc. Natl. Acad. Sci. USA.

[11] Stein JC, Dixit R, Nasrallah ME, Nasrallah JB (1996). SRK, the stigma-specific S locus receptor kinase of *Brassica*, is targeted to the plasma membrane in transgenic tobacco. Plant cell.

[12] Naithani S, Chookajorn T, Ripoll DR, Nasrallah JB (2007). Structural modules for receptor dimerization in the S-locus receptor kinase extracellular domain. Proc. Natl. Acad. Sci. USA.

[13] Tordai H, Banyai L, Patthy L (1999). The PAN module: the N-terminal domains of plasminogen and hepatocyte growth factor are homologous with the apple domains of the prekallikrein family and with a novel domain found in numerous nematode proteins. FEBS Letter.

[14] Loris R (2002). Principles of structures of animal and plant lectins. Biochim. Biophys. Acta.

[15] Herve C (1999). Characterization of the Arabidopsis lecRK-a genes: members of a superfamily encoding putative receptors with an extracellular domain homologous to legume lectins. Plant mol. Biol..

[16] Cambi A, Koopman M, Figdor CG (2005). How C-type lectins detect pathogens. Cell. Microbiol..

[17] Cheng XY (2013). A rice lectin receptor-like kinase that is involved in innate immune responses also contributes to seed germination. Plant J..

[18] Deb S, Sankaranarayanan S, Wewala G, Widdup E, Samuel MA (2014). The S-Domain Receptor Kinase Arabidopsis Receptor Kinase2 and the U Box/Armadillo Repeat-Containing E3 Ubiquitin Ligase9 Module Mediates Lateral Root Development under Phosphate Starvation in *Arabidopsis*. Plant Physiol..

[19] Wan JR (2008). A lectin receptor-like kinase is required for pollen development in *Arabidopsis*. Plant Mol. Biol..

[20] Zuo KJ, Zhao JY, Wang J, Sun XF, Tang KX (2004). Molecular cloning and characterization of GhlecRK, a novel kinase gene with lectin-like domain from *Gossypium hirsutum*. DNA Sequence.

[21] Hirsch AM (1999). Role of lectins (and rhizobial exopolysaccharides) in legume nodulation. Curr. Opin. plant biol..

[22] Navarro-Gochicoa MT (2003). Characterization of four lectin-like receptor kinases expressed in roots of *Medicago truncatula*. Structure, location, regulation of expression, and potential role in the symbiosis with *Sinorhizobium meliloti*. Plant Physiol..

[23] Deng KQ (2009). A Lectin Receptor Kinase Positively Regulates ABA Response During Seed Germination and Is Involved in Salt and Osmotic Stress Response. J.Plant Biol..

[24] Xin ZY, Wang AY, Yang GH, Gao P, Zheng ZL (2009). The Arabidopsis A4 Subfamily of Lectin Receptor Kinases Negatively Regulates Abscisic Acid Response in Seed Germination. Plant Physiol..

[25] Chen XW (2006). A B-lectin receptor kinase gene conferring rice blast resistance. Plant J..

[26] Singh P (2012). The Lectin Receptor Kinase-VI.2 Is Required for Priming and Positively Regulates *Arabidopsis* Pattern-Triggered Immunity. Plant Cell.

[27] Bonaventure G (2011). The Nicotiana attenuata LECTIN RECEPTOR KINASE 1 is involved in the perception of insect feeding. Plant signal. Behav..

[28] Gilardoni PA, Hettenhausen C, Baldwin IT, Bonaventure G (2011). Nicotiana attenuata LECTIN RECEPTOR KINASE1 Suppresses the Insect-Mediated Inhibition of Induced Defense Responses during Manduca sexta Herbivory. Plant Cell.

[29] Wang Y, Nsibo DL, Juhar HM, Govers F, Bouwmeester K (2016). Ectopic expression of Arabidopsis L-type lectin receptor kinase genes LecRK-I.9 and LecRK-IX.1 in *Nicotiana benthamiana* confers Phytophthora resistance. Plant Cell Report.

[30] Singh P, Zimmerli L (2013). Lectin receptor kinases in plant innate immunity. Front. Plant Sci..

[31] Joshi A, Dang HQ, Vaid N, Tuteja N (2010). Pea lectin receptor-like kinase promotes high salinity stress tolerance in bacteria and expresses in response to stress in planta. Glycoconj. J..

[32] He XJ, Zhang ZG, Yan DQ, Zhang JS, Chen SY (2004). A salt-responsive receptor-like kinase gene regulated by the ethylene signaling pathway encodes a plasma membrane serine/threonine kinase. Theor. Appl. Genet..

[33] Liu PL, Du L, Huang Y, Gao SM, Yu M (2017). Origin and diversification of leucine-rich repeat receptor-like protein kinase (LRR-RLK) genes in plants. BMC Evol. Biol..

[34] Liu PL (2016). Duplication and Divergence of Leucine-Rich Repeat Receptor-Like Protein Kinase (LRR-RLK) Genes in Basal Angiosperm *Amborella trichopoda*. Front. Plant Sci..

[35] Fischer I, Dievart A, Droc G, Dufayard JF, Chantret N (2016). Evolutionary Dynamics of the Leucine-Rich Repeat Receptor-Like Kinase (LRR-RLK) Subfamily in Angiosperms. Plant Physiol..

[36] Zan, Y. *et al*. Genome-wide identification, characterization and expression analysis of *Populus* leucine-rich repeat receptor-like protein kinase genes. *BMC Genomics* **14**10.1186/1471-2164-14-318 (2013). 10.1186/1471-2164-14-318 PMC3682895 23663326

[37] Wei Z, Wang J, Yang S, Song Y (2015). Identification and expression analysis of the LRR-RLK gene family in tomato (*Solanum lycopersicum*) Heinz 1706. Genome.

[38] Zhou, F., Guo, Y. & Qiu, L.-J. Genome-wide identification and evolutionary analysis of leucine-rich repeat receptor-like protein kinase genes in soybean. *BMC Plant Biol*. **16**10.1186/s12870-016-0744-1 (2016). 10.1186/s12870-016-0744-1 PMC4776374 26935840

[39] Shumayla *et al*. Genomic Dissection and Expression Profiling Revealed Functional Divergence in *Triticum aestivum* Leucine Rich Repeat Receptor Like Kinases (TaLRRKs). *Front. Plant Sci*. **7**10.3389/fpls.2016.01374 (2016). 10.3389/fpls.2016.01374 PMC5031697 27713749

[40] Sun, X. & Wang, G.-L. Genome-wide identification, characterization and phylogenetic analysis of the rice LRR-Kinases. *Plos One* **6**10.1371/journal.pone.0016079 (2011). 10.1371/journal.pone.0016079 PMC3050792 21408199

[41] Rameneni, J. J. *et al*. Genomic and Post-Translational Modification Analysis of Leucine-Rich-Repeat Receptor-Like Kinases in *Brassica rapa*. *Plos One* **10**10.1371/journal.pone.0142255 (2015). 10.1371/journal.pone.0142255 PMC4654520 26588465

[42] Shumayla, S, S., Pandey, A. K., Singh, K. & Upadhyay, S. K. Molecular Characterization and Global Expression Analysis of Lectin Receptor Kinases in Bread Wheat (*Triticum aestivum*). *Plos One* **11**10.1371/journal.pone.0153925 (2016). 10.1371/journal.pone.0153925 PMC4844157 27111449

[43] Schmutz J (2010). Genome sequence of the palaeopolyploid soybean. Nature.

[44] Tamura K, Stecher G, Peterson D, Filipski A, Kumar S (2013). MEGA6: Molecular evolutionary genetics analysis version 6.0. Mol. Biol. Evol..

[45] Freeling B (2009). in plant gene content following different sorts of duplication: tandem, whole-genome, segmental, or by transposition,” in *Annu*. Rev. Plant Biol..

[46] Hofberger JA, Nsibo DL, Govers F, Bouwmeester K, Schranz ME (2015). A Complex Interplay of Tandem- and Whole-Genome Duplication Drives Expansion of the L-Type Lectin Receptor Kinase Gene Family in the Brassicaceae. Genome Biol. Evol..

[47] Liu, P.-L., Wan, J.-N., Guo, Y.-P., Ge, S. & Rao, G.-Y. Adaptive evolution of the chrysanthemyl diphosphate synthase gene involved in irregular monoterpene metabolism. *BMC Evol. Biol*. **12**10.1186/1471-2148-12-214 (2012). 10.1186/1471-2148-12-214 PMC3518182 23137178

[48] Roy SW, Gilbert W (2006). The evolution of spliceosomal introns: patterns, puzzles and progress. Nat Rev Genet..

[49] Charlotte T (2014). A pair of receptor-like kinases is responsible for natural variation in shoot growth response to mannitol treatment in *Arabidopsis thaliana*. Plant J..

[50] Xin Z, Wang A, Yang G, Gao P, Zheng Z (2009). The *Arabidopsis* A4 subfamily of lectin receptor kinases negatively regulates abscisic acid response in seed germination. Plant Physiol..

[51] Goodstein DM (2012). Phytozome: a comparative platform for green plant genomics. Nucleic Acids Res..

[52] Hall TA (1999). BioEdit: a user-friendly biological sequence alignment editor and analysis program for Windows 95/98/NT. Nucleic Acids Symposium Series.

[53] Krogh A, Larsson B, von Heijne G, Sonnhammer EL (2001). Predicting transmembrane protein topology with a hidden Markov model: application to complete genomes. J. Mol. Biol..

[54] Katoh K, Misawa K, Kuma K-i, Miyata T (2002). MAFFT: a novel method for rapid multiple sequence alignment based on fast Fourier transform. Nucleic Acids Res..

[55] Stamatakis A, Hoover P, Rougemont J (2008). A Rapid Bootstrap Algorithm for the RAxML Web Servers. Syst. Biol..

[56] Hu B (2015). GSDS 2.0: an upgraded gene feature visualization server. Bioinformatics.

[57] Marchler-Bauer A (2011). CDD: a Conserved Domain Database for the functional annotation of proteins. Nucleic Acids Res..

[58] Sigrist CJA (2013). New and continuing developments at PROSITE. Nucleic Acids Res..

[59] Bailey TL (2009). MEME SUITE: tools for motif discovery and searching. Nucleic Acids Res..

[60] Livak KJ, Schmittgen TD (2001). Analysis of relative gene expression data using real-time quantitative PCR and the 2^−ΔΔCT^ method. Methods..

