# Supplementary material for: Duplication and diversification of lectin receptor-like kinases (LecRLK) genes in soybean
Source: Sci Rep. 2018 Apr 12;8:5861. doi: 10.1038/s41598-018-24266-6 (PMC5897391; doi:10.1038/s41598-018-24266-6)
Supplement: Supplementary file 5 — Supplementary Information [file 41598_2018_24266_MOESM5_ESM.pdf]

Duplication and diversification of lectin receptor-like kinases (LecRLK) in soybean, as revealed by evolutionary, structural and expression analysis

Ping-Li Liu[1], Yuan Huang[2], Peng-Hao Shi[1], Meng Yu[1], Jian-Bo Xie[1]*, LuLu Xie[3]*

Supplemental Table S4 Primers for real time PCR.

Primers	sequences
Glyma.16G093900-F	5'-GGTGTAATCTCTGTGAATGA-3'
Glyma.16G093900-R	5'-ATGATGTAGGTTTCAGCG-3'
Glyma.03G079500-F	5'-TGATCTCTTCATTCGCGTGC-3'
Glyma.03G079500-R	5'-AGAGGCAGCTCTTCCAGTTT-3'
Glyma.08G352100-F	5'-TGCCATAGACACCATTAC-3'
Glyma.08G352100-R	5'-ATTACTGTGGATTGAGACTT-3'
Glyma.08G352300-F	5'-AGCCCACACCGAACTTGATA-3'
Glyma.08G352300-R	5'-TTGTGGCTTGGATGGTTTGG-3'
Glyma.13G248800-F	5'-GACTCCACTCAAGGCTCA-3'
Glyma.13G248800-R	5'-ACACCATGCACCCTAATCCA-3'
Glyma.15G065200-F	5'-ATACGAGAGAGAGTTCAGG-3'
Glyma.15G065200-R	5'-CACTGATCCCTATGCTTCG-3'
Glyma.18G185500-F	5'-GCTTCTCTATGCTGGATTC-3'
Glyma.18G185500-R	5'-TAACCTGCTGGAGTCATT-3'
Glyma.07G154100-F	5'-CTGGCGAGGCTCTATAACCA-3'
Glyma.07G154100-R	5'-GGCAACTTCAAGGAGCACAA-3'
Glyma.18G205000-F	5'-TTACCAAGGCCAAGCAAACC-3'
Glyma.18G205000-R	5'-CTCCATCCTAGAAGATAGTG-3'
Glyma.03G051100-F	5'-GCATCAACAGGTAAGAGGAA-3'
Glyma.03G051100-R	5'-AACAGGCACAACAAGGTAA-3'
Glyma.09G072500-F	5'-GGGGAGGTCGCAACTTAGAT-3'
Glyma.09G072500-R	5'-TCACAGTCCTCTCGTGCATT-3'
Glyma.15G181400-F	5'-TCTGCTCCTTCTGCTTGCT-3'
Glyma.15G181400-R	5'-TTGGTCTTGTTAGGGTCCA-3'
